# Efficacious rat model displays non-toxic effect with Korean beechwood creosote: a possible antibiotic substitute

**DOI:** 10.1080/13102818.2014.931696

**Published:** 2014-07-10

**Authors:** Anh Nguyen Thai Quynh, Neelesh Sharma, Kwang Keun Cho, Tae Jong Yeo, Ki Beom Kim, Chul Yon Jeong, Tae Sun Min, Kim Jae Young, Jin Nam Kim, Dong-Kee Jeong

**Affiliations:** ^a^ Faculty of Biotechnology, Jeju National University, Jeju, Korea; ^b^ Department of Animal Resources Technology, Gyeongnam National University of Science and Technology, Jinju, Korea; ^c^ Korea Institute for Animal Products Quality Evaluation, Anyang, Korea; ^d^ Changjobio Research Institute, Changjobio Corporation, Jeju, Korea; ^e^ National Research Foundation (NRF), R&D Policy Team, Daejeon, Korea; ^f^ Swine Science & Technology Center, Gyeongnam National University of Science and Technology, Jinju, Korea

**Keywords:** antibiotic substitute, Korean beechwood creosote, hepatotoxic markers

## Abstract

Wood creosote, an herbal anti-diarrheal and a mixture of major volatile compounds, was tested for its non-toxicological effects, using a rat model, with the objective to use the creosote as an antibiotic substitute. A total of 30 Sprague-Dawley rats were studied to form five groups with 6 rats each. Korea beechwood creosote was supplemented into three test groups with 0.03 g/kg, 0.07 g/kg and 0.1 g/kg body weight/day without antibiotic support, along with a positive control of Apramycin sulphate (at 0.5% of the daily feed) and a negative control. Korean beechwood creosote supplementation showed no negative effect on the body weight gain in comparison to the negative and the positive control groups and the feed conversion ratio was also comparable with that of the control groups. The clinical pathology parameters studied were also under the umbrella of normal range, including liver specific enzymes, blood glucose, total protein, blood urea nitrogen (BUN), which indicated no toxic effect of creosote at the given doses. The non-hepatotoxic effect was also confirmed using hepatic damage specific molecular markers like Tim-p1, Tim-p2 and Tgf-β1. The results suggested that Korean beechwood may be used as antibiotic substitute in weanling pigs feed without any toxic effect on the body. Although the antimicrobial properties of creosote were not absolutely similar to those of apramycin sulphate, they were comparable.

## Introduction

The pig industry in South Korea is well established with 7347 registered pig farms having a sizeable population of 9.88 million pigs.[1] Post-weaning diarrhoea (PWD) leads to serious health and breeding problems, and economic losses for pig farms.[2] In general all infectious diseases, including PWD and associated diseases**,** in weanling pigs have been managed by the use of antibiotic as feed additives since their discovery. Various antibiotics have been used as therapeutic and growth-promoting agents in animal feeds and this has led to overall improvements in the livestock production.[3] However, the development of bacterial resistance [4] and the problem of antibiotic residues in animal products have led to pressure on regulatory authorities and public acuity of the need to ban antibiotics from animal feeds.[5] Like other countries, in Korea, PWD and associated diseases were controlled by adding antibiotic supplementation in the feed, but, after the ban on the use of growth promoting antibiotics by the European Union (EU) in 2006, the Ministry for Food, Agriculture, Forestry and Fisheries, Republic of Korea,[1] has also implemented a ban on the use of growth promoting antibiotics in animal feed since July 1, 2011. Consequently, it increased the interest of researchers to find alternatives to antibiotics, which includes the usage of components such as organic acids, probiotics, plant extracts, etc., with the consideration to improve the healthiness and safety of animal products reaching the consumer. A review of the studies available on the use of antibiotic alternatives in the pig industry reveals that and no study on the toxicological aspect of Korean beechwood has been done to date.

Creosote is the name used for a variety of products that are mixtures of chemicals or herbals. Creosotes are prepared by treating beech and other woods (beechwood creosote) at high temperature. Wood creosote, also referred to as medicinal creosote, is a mixture of major volatile compounds, such as 2-methoxyphenol (guaiacol; 25.2%), 2–methoxy-4-methylphenol (4-methylguaiacol; 21.4%), 3-methylphenol (*m*-cresol; 8.3%), 4-methylphenol (*p*-cresol; 7.9%), 2-methylphenol (*o*-cresol; 4.6%), and phenol (2.8%).[6–8] Wood creosote is a product that is mainly used in the pharmaceutical industry and is distinct from coal tar creosote, which is a known carcinogen.[9] Wood creosote has been used for medicinal purposes throughout the Europe since the early 1800.[10] There are references in the literature which support the use of wood creosote as a herbal anti-diarrhoeal medicine.[10,11] Neonatal diarrhoea is a complex problem resulting from the interaction between one or more infectious agents, immunity and management procedures.[12] *Escherichia coli* has been reported as an important causative bacterial agent of neonatal porcine diarrhoea.[13,14]

Few reports suggest that wood creosote does not have oncogenic properties, which was studied in a long-term safety study in rats.[15] Korean beechwood creosote prepared in the laboratory contains pyroligneous liquor as the principal active ingredient mixed with other herbal ingredients, such as levan, chitosan, alginic acid, locust bean gum, guar gum and wheat flour. Beechwood creosote is also known for qualities like disinfectant, fungicide, laxative activity and in therapeutics leprosy and tuberculosis. Pyroligneous acid contains aldehydes, ketones, di-ketones, esters, alcohols, acids, furan, and pyran derivatives which are produced after the thermal degradation of wood carbohydrates.[16] Pyroligneous acid has been traditionally used as a sterilizing agent, deodorizer, fertilizer, antimicrobial and growth promoting agent. Pyroligneous acid has immense application in industry, agriculture, medicinal and domestic use.[17]

The aim of the present investigation was to assess the effects of Korean beechwood creosote as an antibiotic substitute for weanling pigs with emphasis on the toxicological aspect (if any), in a rat model, so as to search for an alternative to pig feed antimicrobials. Our study was conducted to identify an alternative to antibiotics used in the pig feed industry to maintain the growth performance and control economic losses.

## Materials and methods

### Experimental animals and design

Rats were used as a model animal to study Korean beechwood creosote as an alternative to antibiotics in pig feed. Male SIC: SD Sprague-Dawley rats (four week old) were procured from Japan (SLC, Inc., Japan). Two animals per cage were housed in a standard but controlled temperature and humidity. Standard rat chow (Rat/Mouse Formula 18%, PMI Nutrition, LLC, Brentwood, MD) and sterilized water were provided *ad libitum* to experimental rats. All animals were acclimatized to the animal facility for one week. The experiment was approved by the Animal Ethics Committee of the School of Applied Life Sciences.

A total of 30 rats were divided into five groups with six rats in each group and designated as groups I, II, III, IV and V. In the negative control group I, only rat chow and water were provided without any supplemental antibiotic or creosote. In the positive control group II, the antibiotic apramycin sulphate (Sigma, USA) was supplemented at a rate of 0.5% of the feed. Groups III to V rats were supplemented with Korean beechwood creosote at a rate of 0.03, 0.07 and 0.1 g/kg body weight/day, respectively, without an antibiotic supplement. Creosote and the antibiotic were administered orally (through a 2 mL sterile syringe) daily for four weeks. Korean beechwood creosote was prepared in a laboratory, and contains pyroligneous liquor as the principal active ingredient mixed with other herbal ingredients such as levan, chitosan, alginic acid, locust bean gum, guar gum and wheat flour.

### Body weight gain and feed conversion ratio

The body weight of individual rats was measured on day zero by digital weighing machine and, subsequently, at weekly intervals during the experiment. For the study of effect of creosote supplementation on body weight gain and feed conversion efficiency, regular body weight measurement was done using digital weighing balance at day 0, weeks 1, 2, 3 and 4 of supplementation. The feed conversation ratio (FCR) for each treatment was computed by the following equation [7]:


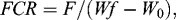


where *F* is the weight of food supplied to a rat during the study period, *W*_0_ is the live weight of a rat at the beginning of the study period and *Wf* is the live weight of a rat at the end of the study period.

### Fecal total Gram-negative bacteria assay

The antibacterial effect of creosote was analysed by counting the total number of intestinal Gram-negative bacteria before and after one week of treatment. About 10 g of feces was collected and homogenized in 100 mL of phosphate buffered saline (PBS, pH 7.0) and different dilutions were made (10^−1^, 10^−2^ and 10^−3^ in PBS) for plating on MacConkey agar. Pink colonies were counted after 12–18 h of incubation at 37 °C. Only those plates which had 30–300 colonies in MacConkey plate were included for the counting. The total number of bacteria was calculated by the following formula:


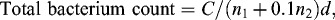


where *C* is the total specific colonies counted in all plates, *n*_1_ is the count on the plates kept for the first dilution, *n*_2_ is the count on the plates kept for the second dilution and *d* is the dilution coefficient correlative to the first dilution.

### The complete blood count (CBC)

About 5 mL of blood samples from the rats of all five groups were collected directly from the heart after the completion of the treatment schedule. Immediately after collection of blood samples, 2 mL of the sample was transferred to sterile screw-capped tubes containing ethylenediaminetetraacetic acid (EDTA) for haematology and the remaining 3 mL was stored in lithium heparinized tubes for clinical biochemistry. Blood samples were analysed using an automatic haematology analyser (Celltak, Hematology Analyzer, Nohon Kohden, Japan) for CBC which includes total white blood cell count (WBC), total red blood cell count (RBC), haemoglobin (Hb), hematocrit test (HCT), mean corpuscular volume (MCV), mean corpuscular haemoglobin (MCH), mean corpuscular haemoglobin concentration (MCHC) and platelet count.

### Blood plasma clinical chemistry

The heparinized samples were centrifuged at 2000*g* for 12 min and supernatant plasma was collected and analysed by an automatic chemistry analyser (Cobas C 111 analyser, Roche). The blood plasma samples were analysed for glucose, cholesterol, blood urea nitrogen (BUN), total protein (TP), albumin, globulin, alanine aminotransferase (ALT) and aspartate aminotransferase (AST).

### Detection of hepatotoxic gene expression

Total RNA was extracted from liver tissues, using an easy-Blue total RNA Extraction Kit (Intron–Biotechnology, Korea) according to the manufacturer's instruction. Concentration and purity was determined by a BioPhotometer machine (Eppendorf – German). The cDNA was then synthesized via reverse transcription (RT) using an oligo-(dT) primer and SuperScript II (Invitrogen). The expression of hepatotoxicity markers such as transforming growth factor beta-1 (Tgfb1), tissue inhibitor of metalloproteinase 1 (Tim-p1) and tissue inhibitor of metalloproteinase 2 (Tim-p2) was detected by reverse transcription polymerase chain reaction (RT-PCR; PCR Master Mix 2x - Promega) with specific primers (Table 1) which were designed by Primer3 software. The housekeeping gene glyceraldehyde 3-phosphate dehydrogenase (GAPDH) was used as an internal control. The PCR amplification protocol was as follows : 95 °C for 2 min followed by 40 cycles of 94 °C for 15 s, 48 °C for 15 s and 72 °C for 20 s. The DNA product was separated by 1% agarose gel electrophoresis and staining with ethidium bromide under ultraviolet light.

**Table 1.** Primer list for amplification of the hepatotoxicity marker.

Gene	Gene bank accession	Sense primer (5′–3′)	Antisense primer (5′–3′)
TIMP-1	NM_053819.1	GCACAGTGTTTCCCTGTTCA	GTCATCGAGACCCCAAGGTA
TIMP-2	NM_021989.2	AAGATCACACGCTGCCCTAT	GGGTCCTCGATGTCAAGAAA
TGF-в1	NM_021578.2	CAATGGGATCAGTCCCAAAC	TTCTCTGTGGAGCTGAAGCA
GAPDH	NM_017008.3	TGGAGTCTACTGGCGTCTT	TGTCATATTTCTCGTGGTTCA

### Statistical analysis

Values of coliform numbers obtained were corrected using a log_10_ transformation before analysis. Analysis of variance (ANOVA) technique was applied to test the significance of the differences between the results obtained with different treatments and also Student's *t*-test was used.

## Results and discussion

### Therapeutic effect of creosote on body weight gain

**Figure 1.**
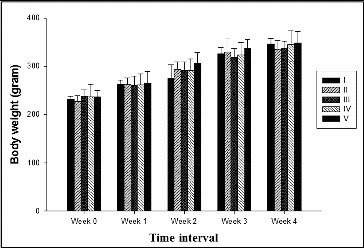
Effect of Korean beechwood creosote on body weight gain at different time intervals.

**Figure 2.**
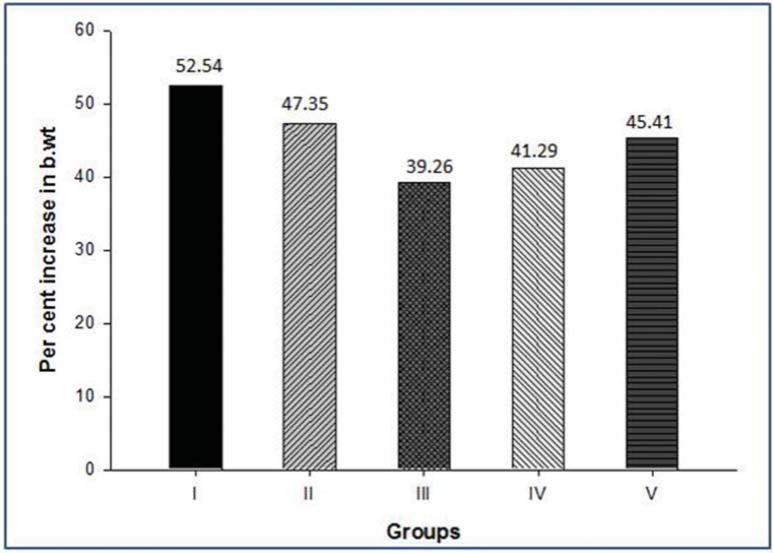
Per cent increase in body weight in four weeks (after completion of supplementation).

Individual animal body weight was recorded every week from day 0 till completion of treatment. The growth rate of the treated groups were compared with the negative (Group I) and positive control (Group II) groups to study the toxic effect of creosote based on the body weight gain. Korean beechwood creosote supplementation did not show any negative effect on the body weight gain in comparison to the negative and positive control groups. The body weight gain of creosote treated animals was similar to that of the control groups (Figure 1). However, the per cent body weight gain was non-significantly increased within the creosote supplemented groups, in a dose-dependent manner from 0.03 to 0.1 g/kg body weight/day (Figure 2). It suggests that Korean beechwood creosote has growth-promoting activity, which could be supported by further evidence after a large-scale experimental trial in pigs as model animals. The feed conversion ratio among creosote treated groups had comparable values with the control groups (Table 2).

**Table 2.** Effects of supplement on performance of rats (mean ± SD).

Index	Group I	Group II	Group III	Group IV	Group V
Initial weight (g)	229.77 ± 4.80	226.43 ± 13.53	242.03 ± 7.33	242.43 ± 28.65	234.37 ± 14.66
Final weight (g)	350.50 ± 10.40	333.65 ± 19.68	337.05 ± 11.20	342.53 ± 33.03	340.80 ± 17.92
Weight gain (g)	114.75 ± 14.58	108.89 ± 9.44	99.09 ± 9.50	108.31 ± 28.29	102.76 ± 19.05
Feed intake (g)	146.06 ± 26.00	134.31 ± 39.62	128.24 ± 29.51	139.07 ± 15.07	147.37 ± 34.15
Feed conversion	1.28 ± 0.15	1.24 ± 0.15	1.30 ± 0.10	1.30 ± 0.18	1.44 ± 0.09

### Fecal total Gram-negative bacterial count

Almost 90%–95% of Gram-negative bacteria are considered to be harmful for the host. In regard to the antimicrobial property of Korean beechwood, we considered the fecal total Gram-negative bacterial count before and after one week of treatment. Creosote treated groups showed non-significant decrease in the total bacterial count after one week of treatment in comparison to the negative control. This indicates that Korean beechwood creosote possesses antibacterial activity. However, the total Gram-negative bacterial count was significantly (*P* < 0.05) reduced in the antibiotic supplemented group after one week of treatment (Table 3) and the antibiotic group values were comparable to those of the creosote treated groups.

**Table 3.** Fecal total Gram-negative bacterial count before and after one week of treatment (mean ± SD).

	Total coliforms count (log_10_ CFU/mL)				
Time duration	Group I	Group II	Group III	Group IV	Group V
Before treatment	5.26 ± 1.76^a^	6.91 ± 0.52^a^	5.41 ± 1.24^a^	5.69 ± 1.25^a^	5.65 ± 1.21^a^
After one week	5.27 ± 0.43^a^	4.23 ± 0.6^b^	5.04 ± 0.29^a^	4.99 ± 0.39^a^	5.29 ± 0.22^a^

Note: Mean values with different superscripts within a column differ significantly (*P* < 0.05).

The antimicrobial activity of Korean beechwood might be attributed to the presence of pyroligneous acid. It was reported that the antimicrobial activity of pyroligneous acid is due to the presence of compounds such as phenolic compounds, carbonyls and organic acids.[18] Phenolic compounds, and in particular methoxyphenols, have been considered as the major contributors to smoke aroma and are responsible for the antimicrobial effects.[19–21] However, Ataka et al. [22] have reported that when wood creosote (Seirogan) was administered orally, the intraluminal concentration of wood creosote was not enough to achieve this microbicidal effect. Through further animal tests, they have shown that antimotility and antisecretory actions are the principal antidiarrheal effects of wood creosote. Wood creosote inhibits intestinal secretion induced by enterotoxins by blocking the Cl^−^ channels on the intestinal epithelium.[22] As wood creosote has a long history as a known gastrointestinal microbicidal agent, we expect that the antibacterial activity of Korean beechwood may be increased by using creosote at higher doses.

### Complete blood count (CBC)

Table 4 indicates the values of hemogram obtained, which showed a similar pattern to those in the negative control and the positive control, which suggests no negative impact of Korean beechwood creosote on the hemogram. The white blood cell count (WBC) was 12.12 × 10^3^/μL to 12.75 × 10^3^/μL in creosote treated animals, which was below the range of the negative and the positive control groups (12.59 × 10^3^/μL and 12.27 × 10^3^/μL, respectively). The total red blood cells (RBCs) count and haemoglobin also indicated the healthy status of the treated animals was like that of the negative control animals.

**Table 4.** Effect of Korean beech wood Creosote on blood hemogram (mean ± SD).

Blood hemogram	Group I	Group II	Group III	Group IV	Group V
WBC (10^3^/μL)	12.59 ± 2.76	12.27 ± 2.66	12.14 ± 2.20	12.12 ± 2.03	12.75 ± 2.72
RBC (10^6^/μL)	7.87 ± 0.45	7.89 ± 0.68	7.82 ± 0.60	7.78 ± 0.44	7.72 ± 0.58
Hb (g/dL)	15.70 ± 0.92	16.14 ± 1.28	15.22 ± 1.63	15.53 ± 1.00	15.46 ± 1.00
HCT (%)	47.35 ± 2.24	48.56 ± 2.04	47.65 ± 1.25	47.10 ± 0.91	47.11 ± 1.49
MCV (fL)	60.28 ± 3.55	61.88 ± 4.64	61.46 ± 4.42	60.68 ± 3.72	61.34 ± 4.93
MCH (pg)	19.96 ± 0.79	20.48 ± 0.46	19.41 ± 0.67	19.94 ± 0.42	20.03 ± 0.56
MCHC (g/dL)	33.15 ± 1.23	33.24 ± 2.22	31.90 ± 2.85	32.97 ± 2.15	32.85 ± 2.61
Platelets (10^3^/μL)	121.15 ± 137.63	118.05 ± 117.95	120.30 ± 156.36	124.25 ± 171.31	121.37 ± 125.13

### Blood plasma chemistry

Altered blood serum/plasma biochemistry is an important indicator of toxicity in the body. The blood plasma samples of the negative and positive control and of the creosote treated groups were subjected to various biochemical examinations to discern the toxic effect of creosote.

The results indicated that the biochemical analysis of creosote treated animals showed no significant difference in the blood biochemical profile, including liver damage indicators (liver specific enzymes, albumin etc.) and kidney damage indicator (e.g. total protein, BUN), and even all metabolic byproducts including glucose, cholesterol and protein were also within the normal range as in the control group. The values in the creosote treated groups were similar to those in the antibiotic treated animals. Table 5 shows important blood metabolites including glucose, cholesterol, BUN, total protein, albumin, globulin, ALT and AST in all groups. The blood plasma glucose level in creosote treated animals was in the normal range (50–160 mg/dL). These results potentially indicate that Korean beechwood creosote does not have a hypo- and/or hyper-glycemic effect on the body, and is safe within the given doses and duration of treatment. Plasma ALT is a liver specific injury indicator, which was also recorded to be within the normal range in the creosote treated groups during the treatment period. The blood plasma protein level, particularly that of albumin, was also within the normal values; however, reduced albumin level is a good indicator of hepatic toxicity.

**Table 5.** Effect of Korean beech wood creosote on blood plasma biochemical constituents (mean ± SD).

Blood plasma constituents	Group I	Group II	Group III	Group IV	Group V
Glucose (mg/dL)	153 ± 28.34	151 ± 19.19	150 ± 26.16	147 ± 10.25	147 ± 9.28
Cholesterol (mg/dL)	81.83 ± 4.79	80.33 ± 9.50	68.33 ± 12.16	70 ± 7.59	77.67 ± 12.89
BUN (mg/dL)	15.50 ± 3.39	14.00 ± 2.68	15.17 ± 2.48	14.17 ± 0.98	14.83 ± 1.94
Total protein (g/dL)	5.53 ± 0.44	5.65 ± 0.24	5.40 ± 0.37	5.13 ± 0.37	5.52 ± 0.59
Albumin (g/dL)	4.07 ± 0.23	4.15 ± 0.29	3.75 ± 0.27	3.68 ± 0.29	3.80 ± 0.24
Globulin (g/dL)	1.47 ± 0.26	1.50 ± 0.11	1.65 ± 0.18	1.45 ± 0.23	1.72 ± 0.39
ALT (U/L)	67.67 ± 26.51	58.83 ± 12.78	59.33 ± 14.72	49.17 ± 6.11	51.17 ± 8.47
AST (U/L)	115.67 ± 42.97	88.67 ± 13.43	113.17 ± 39.93	80.83 ± 12.43	92.67 ± 34.06

In respect to the toxicological effect of Korean beechwood creosote on the studied hematobiochemical values in creosote treated animals, all hematobiochemical parameters were within the normal range, which indicated no negative impact on haematology. Kuge et al. [15] have also reported no changes in the clinical pathology in rats treated with 20–200 mg/kg body weight/day. Liver and kidney are the major organs of the body that metabolize and excrete the byproducts/toxic substances from the body; hence both are good indicator of drug toxicity. Liver injury can be diagnosed by certain biochemical markers like ALT, AST, total protein, albumin, etc. Elevations in serum enzyme levels in these markers can be taken as the relevant indicators of liver toxicity. ALT is considered a more specific and sensitive indicator of hepatocellular injury than AST. In contrast, our study has all the studied enzymological and biochemical values within the normal range. Normal values of BUN in our study also indicated no toxic effect on the kidneys and also supported the evidence of non-toxic effect on the liver. The BUN level increases in case of improper kidney functioning, while BUN becomes low when there is liver damage.

### Detection of hepatotoxic gene expression

**Figure 3.**
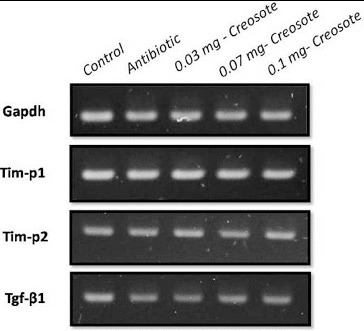
RT-PCR detection of the expression of liver damage-related marker in rat liver tissues. GAPDH was used as an internal control.

After the completion of supplementation, animals were euthanized and liver tissue was collected for a hepatotoxic study. The RT-PCR using primers of hepatic damage indicator genes like transforming growth factor beta-1 (Tgfb1), tissue inhibitor of metalloproteinase 1 (Tim-p1) and tissue inhibitor of metalloproteinase 2 (Tim-p2) was performed to study the hepatotoxicity at the molecular level. In our study, Tim-p1 expression was similar in all creosote treated groups to that in the control groups. Non-significant down-regulation of the Tim-p2 and Tgf-β1 genes was recorded with increasing the concentration of creosote from 0.03 mg to 0.1 mg (Figure 3). The down-regulation of Tgf-β1 indicated the hepatoprotective role of Korean beechwood creosote. These results strongly suggested that creosote does not have hepatotoxicity up to a dose rate of 0.1 mg/kg body weight in rats.

In the present experiment, the toxicological study at the molecular level also suggested a non-toxic effect of Korean beechwood at the given dose, which was evidenced by the expression profile of the selected hepatotoxic markers such as Tim-p1, Tim-p2 and Tgf-β1 genes in the liver tissue of all groups. Previous biochemical studies have identified various well-known hepatic damage markers.[23–25] Tim-p1, Tim-p2 and Tgf-β1 mRNA expression became up-regulated in hepatic toxicity [21] but this study showed a similar expression pattern to that in the healthy control group. However, Tim-p2 and Tgf-β1 expression was down-regulated with increasing the dose of creosote, which indicated that Korean beechwood is likely to have a hepatoprotective effect. Su et al. [26] have also found that the hepatoprotective agent (Silymarin) caused down-regulation of Tgf-β1.

Creosotes are complex mixtures of variable composition and the individual components are likely to show interspecies variation in toxicity. Owing to their diversity, variability in biological effect, and composition, it is nearly impossible to define a general mechanism of toxicity for creosotes. Basic and toxicological information on individual components is cannot serve to adequately define the properties of the whole mixture and for this reason no physiologically based pharmacokinetic (PBPK)/pharmacodynamic (PD) models have been proposed for creosote.

Korean beechwood creosote mainly consists of simple phenolic compounds, and guaiacol, creosol and 4-ethylguaiacol.[27] Beechwood creosote has been used as an anti-diarrheal agent [28,29] and also in the treatment of leprosy, pneumonia, and tuberculosis in human medicine. It has been noted that beechwood creosote at the rate of 394 mg/kg/day in the diet for 96 weeks induced the signs of toxicity in rats, but failed to produce treatment-related increase in the incidence of tumours.[9]

Sub-therapeutic levels of antimicrobial feed additives have been used in swine feeds since the 1950s for improved growth rate and feed efficiency and to maintain pig performance in the presence of sub-clinical disease.[30] Apramycin was first approved in the United States in 1986 for the treatment and prevention of bacterial infections associated with weaning in livestock.[31] Aparamycin sulphate is used as feed additive for the control of bacterial enteritis in weanling pigs due to *E. coli* and other associated factors, but recently the use of antibiotics in the feed has been banned. Resistance to this antibiotic was subsequently shown to occur in various bacteria.[12,31–33] The most important thing is the development of antibiotic resistance, raised the serious question for consumers health and its considered as a global ecological problem.[31] The normal intestinal microfloras of animals represent a reservoir of antibioresistant strains and/or antibioresistance genes, that can be transmitted to humans.[34] All these issues increased the interest of authors to identify alternatives to antibiotics use in the pig feed industry to maintain growth performance.[35] Recently, other researchers have been tried to replace the antibiotics in pig feed with potato protein [36] and wood vinegar.[37]

## Conclusions

Based on the results of the present study, it was concluded that Korean beechwood may be used as an antibiotic substitute for the control of diarrhoea in pig industry without any toxic effect on the animal body. We found all hematological and plasma blood chemistry values were within the normal range, which indicated no negative and/or toxic effect of Korean beechwood creosote on the animal body. Administration of creosote at different concentrations tested had no effect on liver-specific enzymes. The present findings also reported the non-toxic effect on kidney functioning.
